# Imaging the PM/AICD patient; is it evolving into a routine procedure? An evolutionary report of our first 100 patients

**DOI:** 10.1186/1532-429X-17-S1-T8

**Published:** 2015-02-03

**Authors:** June A Yamrozik, Mark Doyle, Ronald B Williams, Geetha Rayarao, Diane V Thompson, Moneal Shah, Huma Samar, Robert W Biederman

**Affiliations:** 1 Cardiac MRI, Allegheny General Hospital, Pittsburgh, PA, USA; 2 Cardiology, VA Medical Center, Los Angeles, CA, USA

## Background

Pacemaker/AICD use may no longer be prohibitive in the MRI environment. Seminal work by us and others has pointed towards increasing safety and specifically, the marked additive clinical value. Historically, on only extraordinarily high-risk patients with acute diagnoses were imaged. However, over time, we began to note an interesting trend as our understanding, science and comfort level admixed with zero-event rate almost imperceptibly causes us to ‘relax' the mandate for acuity. We wondered, "Has imaging a patient with a pacemaker that was once considered a last resort procedure started to evolve into a routine study? What have we learned from performing these procedures?

### Hypothesis

We hypothesize that there may be a learning curve imaging patients with pacemakers?

## Methods

We tracked the indication and matched that to the date and exam performed grouped into annual increments of our PM/CMR over 10years. Unchanged commonalities included scanner (GE 1.5T, Milwaukee, WI), 2 technologists and CMR MD. ‘Acute' meaning life endangering diagnosis/presentation was established by agreement between the scanning technologist and the MD. A total of 100 patients were imaged on a GE CV/i Excite Version 12, 1.5 T CRM (GE, Milwaukee, WI). 60 patients had a complete pacemaker implantation, 17 patients had an AICD, 10 patients had an AICD/Pacemaker, 5 patients had a single pacemaker lead, 8 REVO pacemakers (1 not approved for Cardiac pacemaker imaging). The cardiologist had the final word after conversing with the ordering physician if the patient warranted this procedure. All patients signed informed consent.

## Results

**Figure 1:**
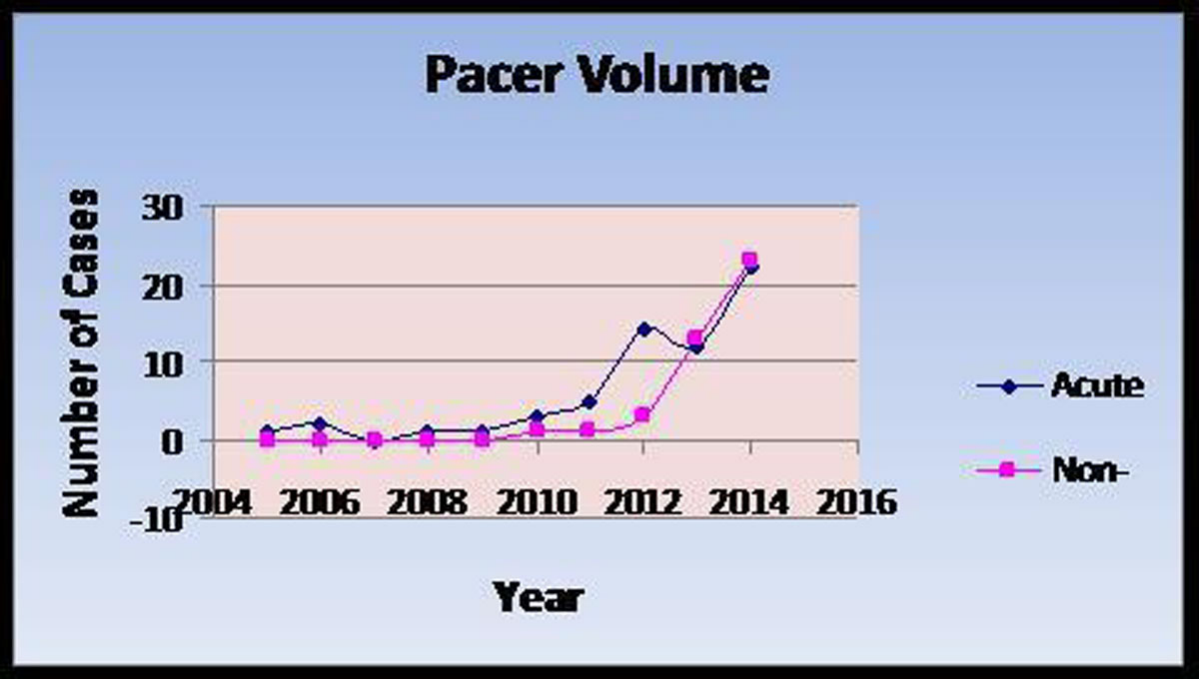


A total of 100 patients with implanted PM, AICD's or combination underwent scanning. During the early stages (2005-2011), a total of 15 patients completed this procedure with no adverse events all with critical need for a CMR. In 2012: 17pts, 2013: 25pts, and in 2014: 43pts underwent scanning. In 2014 the number almost doubled to 43 patients and in some instances was considered the first option to get the best possible diagnosis. (Figure 1). Examining the temporal acuity relationship, we observed a similar relationship. Namely, with time, the indication innocuously expanded such that proportionately, non-life threatening cases comprised an increasing population

## Conclusions

What was once considered a last resort procedure is now becoming a more routine event. With confidence in performing CMR-PM/AICD imaging, along with a zero adverse event, has led to expanded indications for patients potentially candidates for imaging. No longer is the bar set so high that only patients with acute diagnoses are being scanned but rather non-acute more routine diagnoses are being imaged suggesting there *is* a learning curve.

## Funding

Internal.

